# Pharmacogenomics assists in controlling blood pressure in cardiovascular and cerebrovascular patients during Rehabilitation: a case report

**DOI:** 10.3389/fphar.2024.1424683

**Published:** 2024-10-08

**Authors:** Tian Hou, Luhai Yu, Xiaoliang Shi, Yueran Zhen, Longyu Ji, Zhenbang Wei, Yipeng Xu

**Affiliations:** ^1^ Department of Rehabilitation, People’s Hospital of Xinjiang Uygur Autonomous Region, Urumqi, Xinjiang, China; ^2^ Department of Medical Scientific Affairs, WuXi Diagnostics Innovation Research Institute, Shanghai, China

**Keywords:** pharmacogenomics, hypertension, precise diagnosis and treatment, control blood pressure, case report

## Abstract

Hypertension is a common risk factor for cardiovascular disease. Pharmacogenomics, as a tool for personalized healthcare, helps in determining the optimal drug treatment based on the genome of individual patient. This study reports a 49-year-old male with acute cerebral infarction, pulmonary infection, extremely high-risk hypertension (grade3), type 2 diabetes, hyperhomocysteinemia, hyperlipidemia, and fatty liver. The patient initially received conventional systemic treatment but continued to have severe hypertension (159/85 mmHg). To better control blood pressure, a pharmacogenomic test was performed, and results showed that the SNP genotype of rs4961 (*ADD1*) suggests poor efficacy with certain antihypertensive drugs. The genotype of rs4149601 (*NEDD4L*) indicates better efficacy with hydrochlorothiazide, while the CYP3A5*3 genotype indicates a slow metabolism of calcium channel blockers, suggesting that amlodipine may be more effective than nifedipine. By replacing nifedipine with amlodipine and increasing the dosage of hydrochlorothiazide, the patient’s systolic blood pressure was stabilized, although diastolic blood pressure remained suboptimal (131/91 mmHg). Despite low potassium levels, the patient was not sensitive to spironolactone (141/91 mmHg) but achieved exhibited well-controlled blood pressure (129/90 mmHg) with hydrochlorothiazide, consistent with pharmacogenomics recommendations. In summary, pharmacogenomics testing identified genetic variations influencing the patient’s response to specific drugs, guiding their selection and administration. This approach can lead to better blood pressure control and reduce the risk of adverse drug events, highlighting the potential of personalized drugs in managing hypertension through pharmacogenomics.

## Introduction

Cardiovascular disease is the leading global cause of death, accounting for nearly 32% of all deaths worldwide (Tsao et al., 2023; Roth et al., 2020). Hypertension, a major risk factor for both cardiovascular and cerebrovascular events (GBD 2017 Risk Factor Collaborators, 2018), is the most common chronic noninfectious disease, with a high incidence rate (Mills et al., 2020). As of 2023, hypertension affects approximately 1.28 billion people globally (WHO, 2023), with projections suggesting that this number will exceed 1.5 billion by 2025 (Kearney et al., 2005). Inadequate blood pressure control remains a significant clinical challenge for many hypertensive patients (Choudhry et al., 2022; Oparil et al., 2018), leading to organ damage, stroke, kidney disease, and other cardiovascular conditions, which in turns greatly increases economic and social costs (Zhou et al., 2018; De Bhailis and Kalra, 2022).

Established non-pharmacological interventions for preventing and treating hypertension include weight loss, reducing dietary sodium, increasing potassium intake, following a heart-healthy diet, engaging in physical activity, and reducing alcohol consumption (Verma et al., 2021). First-line pharmacologic therapy for hypertension involves thiazide diuretics, calcium channel blockers, angiotensin-converting enzyme inhibitors, or angiotensin receptor blockers, and sometimes a combination of two of these drugs (Wright et al., 2018; Carey et al., 2022). It is important to note that angiotensin-converting enzyme inhibitors and angiotensin receptor blockers should not be administered simultaneously. However, many patients struggle with drug adherence due to adverse reactions, contributing to increasing incidence of uncontrolled hypertension (Kvarnström et al., 2021). Tailored precise medication treatment for hypertensive patients can significantly reduce the societal burden of disease by improving the safety and efficacy of antihypertensive drugs.

Pharmacogenomics decision-making aids clinicians in making informed decisions by utilizing clinically actionable genetic variations in pharmacokinetic and pharmacodynamic genes that affect drug safety, tolerance, and response. Numerous studies have highlighted the advantages of pharmacogenomics in hypertension treatment, including research such as the Genetics of Hypertension Associated Treatments (GenHAT) and the genetics of drug response in primary hypertension (GENRES) (Armstrong et al., 2022; McDonough et al., 2018). However, the application of pharmacogenomics in hypertension in China remains underreported, therefore meaningful case studies can significantly contribute to this field.

This case report highlights the importance of pharmacogenomics in clinical practice by presenting a case of a Chinese hypertensive patient who successfully managed his hypertension through pharmacogenomics-guided treatment.

## Case presentation

A 49-year-old Han male patient with primary hypertension, who has had a history of hypertension for over 9 years, reached a maximum hypertension of 170/120 mmHg without taking any antihypertensive medication. The patient information, medical records, and blood pressure outcomes in this case report were collected from the Department of Rehabilitation at the People’s Hospital of Xinjiang Uygur Autonomous Region. This case study was approved by the Ethics Committee of People’s Hospital of Xinjiang Uygur Autonomous Region, and written informed consent was obtained from the patient before conducting cardiovascular pharmacogenomic (PGx) testing.

On 11 May 2022, the patient was admitted with sudden onset of left limb weakness and was diagnosed with cerebral infarction. At the time of admission, his blood pressure was 207/135 mmHg on the left and 182/124 mmHg on the right. Blood lipid levels were as follows: triglycerides at 3.86 mmol/L, total cholesterol at 5.56 mmol/L, high-density lipoprotein cholesterol at 1.26 mmol/L, low-density lipoprotein cholesterol at 2.62 mmol/L, and an arteriosclerosis index of 3.41. The patient was diagnosed with acute cerebral infarction, pulmonary infection, extremely high-risk hypertension (grade 3), type 2 diabetes, hyperhomocysteinemia, hyperlipidemia, and fatty liver.

The patient initially received intravenous thrombolysis with ateplase (7 mg), but the treatment was terminated due to significant blood pressure fluctuations. Subsequently, butylphthalide was administered to improve collateral circulation. Nifedipine, sacubitril valsartan, and metoprolol tartrate were prescribed for blood pressure reduction, insulin pump therapy for blood sugar control, clopidogrel for anti-aggregation, and atorvastatin for lowering blood lipids. The patient’s symptoms improved significantly after treatment, and by discharge, blood pressure was controlled at 132/105 mmHg. The patient was advised to continue with clopidogrel (75 mg, once daily) for long-term anti-thrombotic therapy, atorvastatin calcium tablets (20 mg, once per night) for long-term anti-atherosclerotic treatment, insulin glargine (14U) combined with metformin (500 mg, three times daily) for long-term hypoglycemic treatment, along with sacubitril valsartan sodium tablets (100 mg, twice daily), metoprolol target tablets (25 mg, twice daily), and nifedipine controlled-release tablets (30 mg, once daily) for long-term treatment of blood pressure management.

**FIGURE 1:**
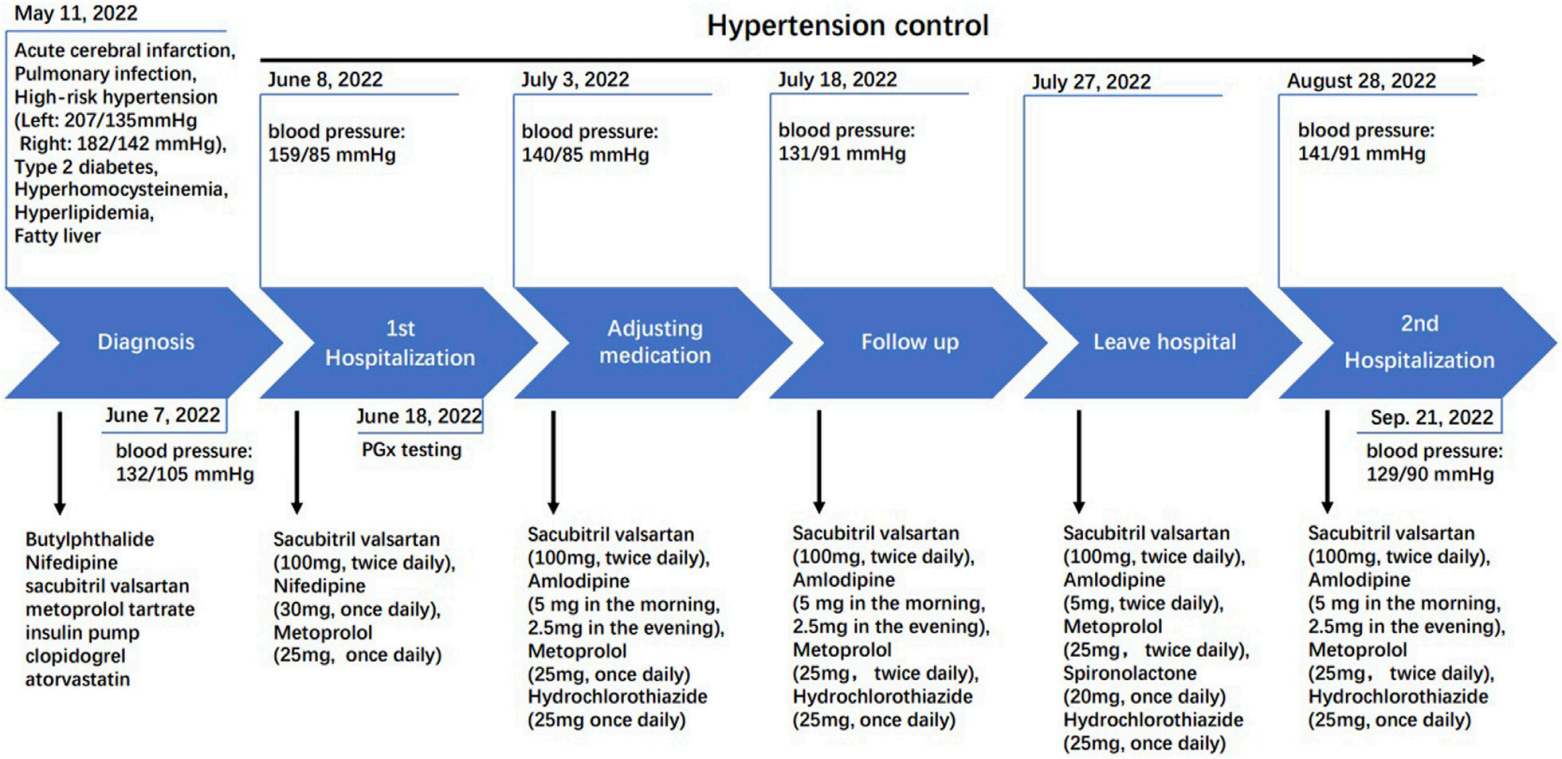
Clinical history of the patient in hypertension controlling.

On 8 June 2022, the patient’s blood pressure test (159/85 mmHg) indicated unstable blood pressure with elevated systolic levels (Figure 1). We subsequently recommended a pharmacogenomics testing package, utilizing a time-of-flight mass spectrometry platform provided by Wuxi Diagnostic Company. This panel includes 54 SNPs across 26 genes and structural variations of CYP2D6, selected based on the CPIC guideline, PharmGKB database and some Chinese cardiovascular disease treatment official guidelines, with consideration for polymorphisms in the Chinese population. The panel covers β-receptor blockers such as metoprolol, angiotensin receptor blockers like valsartan, calcium channel blockers such as amlodipine and nifedipine, and diuretics including spironolactone and hydrochlorothiazide.

The pharmacogenomics test results showed that the C/C genotype of rs1801253 in *ADRB1* supports the normal efficiency of metoprolol (Parvez et al., 2012). The presence of 11 SNPs (rs1065852, rs1135822, rs1135840, rs16947, rs28371725, rs35742686, rs3892097, rs5030655, rs5030865, rs72549349, rs72549352) in the *CYP2D6* suggests normal metabolism of β-receptor blockers (Thomas and Johnson, 2020). The G/G genotype of rs1283807 indicates a relatively good efficiency of valsartan (Kamide et al., 2013), while the C/C genotype of rs776746 (*CYP3A5*), corresponding to a *3/*3 genotype in the patient, supports slower metabolism of calcium channel blocker drugs such as amlodipine and nifedipine (Park et al., 2006). The G/T genotype of rs4961 (*ADD1*) suggests reduced efficacy of spirionolactone (Thorn et al., 2013), and the G/A genotype of rs4149601 (*NEDD4L*) supports better efficacy of diuretics such as hydrochlorothiazide (Luo et al., 2009; Manunta et al., 2008). These findings are detailed in Table 1.

**TABLE 1:** The results of pharmacogenomic testing for the patient.

Gene	SNP location	Genotype	Description	Phenotype
*ABCB1*	rs1045642	G/A	Heterozygous	—
*ABCB1*	rs2032582	C/A	Heterozygous	—
*ABCC9*	rs1283807	G/G	Mutant	—
*ACE*	rs4291	A/A	Mutant	—
*ACE*	rs4343	A/A	Mutant	—
*ADD1*	rs4961	G/T	Mutant	—
*ADRB1*	rs1801253	C/C	Mutant	—
*AGT*	rs5051	T/T	Mutant	—
*AGTR1*	rs5186	A/A	Wild type	—
*APOE*	rs429358	T/T	Wild type	—
*APOE*	rs7412	C/C	Wild type	—
*BDKRB2*	rs8012552	C/C	Wild type	—
*CACNA1C*	rs1051375	A/A	Mutant	—
*CACNA1C*	rs2238032	T/T	Wild type	—
*CES1*	rs2244613	G/T	Heterozygous	—
*CES1*	rs8192935	A/G	Heterozygous	—
*CYP2C19*	rs12248560	C/C	Wild type	NM
*CYP2C19*	rs12769205	A/A	Wild type	NM
*CYP2C19*	rs28399504	A/A	Wild type	NM
*CYP2C19*	rs3758581	G/G	Mutant	NM
*CYP2C19*	rs4244285	G/G	Wild type	NM
*CYP2C19*	rs4986893	G/G	Wild type	NM
*CYP2C19*	rs72552267	G/G	Wild type	NM
*CYP2C9*	rs1057910	A/A	Wild type	NM
*CYP2C9*	rs1799853	C/C	Wild type	NM
*CYP2D6*	rs1065852	G/G	Wild type	NM
*CYP2D6*	rs1135822	A/A	Wild type	NM
*CYP2D6*	rs1135840	C/C	Wild type	NM
*CYP2D6*	rs16947	G/G	Wild type	NM
*CYP2D6*	rs28371725	C/C	Wild type	NM
*CYP2D6*	rs35742686	T/T	Wild type	NM
*CYP2D6*	rs3892097	C/C	Wild type	NM
*CYP2D6*	rs5030655	A/A	Wild type	NM
*CYP2D6*	rs5030865	C/C	Wild type	NM
*CYP2D6*	rs72549349	C/C	Wild type	NM
*CYP2D6*	rs72549352	—	Wild type	NM
*CYP3A4*	rs2242480	C/C	Wild type	—
*CYP3A4*	rs35599367	G/G	Wild type	—
*CYP3A5*	rs776746	C/C	Mutant	—
*CYP4F2*	rs2108622	C/T	Heterozygous	IM
*GNB3*	rs5443	T/T	Mutant	—
*KCNJ1*	rs11600347	C/C	Wild type	—
*MTHFR*	rs1801133	A/G	Heterozygous	—
*NEDD4L*	rs4149601	G/A	Heterozygous	—
*PEAR1*	rs12041331	G/G	Wild type	—
*PTGER3*	rs11209716	T/T	Wild type	—
*PTGS1*	rs10306114	A/A	Wild type	—
*SLCO1B1*	rs2306283	G/G	Mutant	—
*SLCO1B1*	rs4149015	G/A	Heterozygous	—
*SLCO1B1*	rs4149036	C/A	Heterozygous	—
*SLCO1B1*	rs4149056	T/C	Heterozygous	—
*VKORC1*	rs7294	T/C	Heterozygous	—
*VKORC1*	rs9923231	C/T	Heterozygous	—
*VKORC1*	rs9934438	G/A	Heterozygous	—

On 3 July 2022, the patient’s blood pressure was 140/85 mmHg. Based on the results of pharmacogenomic testing, the patient’s medication was adjusted, replacing nifedipine with a combination of amlodipine and hydrochlorothiazide. The adjusted medication regimen included sacubitril valsartan sodium tablets (100 mg, twice daily), metoprolol tartrate tablets (25 mg, once daily), amlodipine besylate tablets (5 mg in the morning and 2.5 mg in the evening), and hydrochlorothiazide tablets (25 mg, once daily) (Figure 1). By 18 July 2022, the patient’s blood pressure had been controlled to 131/91 mmHg. Given the slightly elevated diastolic blood pressure, which may be associated with a low potassium level (3.6 mmol/L, Table 2), the dosage of amlodipine was increased, and a small dose of diuretic spironolactone was added on 18 July 2022. The revised medication list for blood pressure management was: sacubitril valsartan sodium tablets (100 mg, twice daily), metoprolol tartrate tablets (25 mg, twice daily), amlodipine besylate tablets (5 mg, twice daily), spironolactone (20 mg, once daily, with a typical dose of 40–80 mg daily), and hydrochlorothiazide tablets (25 mg, once daily) (Figure 1).

**TABLE 2:** The list of related biomedical parameters of the patient during the process of blood pressure control.

Biochemical parameters	Reference range	Time		
		06/9/2022	07/18/2022	08/28/2022
Triglycerides (mmol/L)	<1.7	1.93	3.39	2.47
Total cholesterol (mmol/L)	2.59–6.47	2.9	3.97	3.45
HDL (mmol/L)	>1.04	0.75	0.99	0.95
LDL (mmol/L)	0–3.37	1.54	2.15	1.8
Atherosclerosis index	<4	2.87	3.01	2.63
Creatine kinase	24–194	65	57	74
Potassium (mmol/L)	3.5–5.3	3.7	3.6	3.4
Sodium (mmol/L)	137–147	139	144	143
Total serum bile acids (TSBA, μmol/L)	0–10.0	2.4	3	1.3

By 28 August 2022, a follow-up examination revealed an increase in blood pressure (141/91 mmHg). Blood lipid test showed triglycerides at 2.47 mmol/L, total cholesterol at 3.45 mmol/L, high-density lipoprotein cholesterol at 0.95 mmol/L, low-density lipoprotein cholesterol at 1.80 mmol/L, and an arterial sclerosis index of 2.63. Notably, the patient’s potassium and sodium levels were 3.4 mmol/L (reference range: 3.5–5.3 mmol/L) and 143 mmol/L (reference range: 137–147 mmol/L), respectively (Table 2). Given the poor efficacy of spironolactone as indicated by pharmacogenomics testing, spironolactone was discontinued, and the dose of amlodipine was reduced (5 mg in the morning and 2.5 mg in the evening). By 21 September 2022, blood pressure had been controlled at 129/90 mmHg (Figure 1). The patient was informed by phone in January 2023 that his blood pressure remained relatively stable, and he did not return to the hospital for further follow-up.

## Discussion

Uncontrolled hypertension is a crucial factor in the treatment of cardiovascular diseases (Whelton et al., 2018). A 2018 study revealed that more than half of hypertensive patients had difficulty in maintaining their blood pressure below 140/90 mmHg (Muntner et al., 2020), highlighting the need for medication treatment for patients with blood pressure exceeding this threshold (Joint Committee for Guideline Revision, 2019). Despite improvements in hypertension management over the past two to three decades, it continues to pose a major global health threat (NCD Risk Factor Collaboration NCD-RisC, 2021), with rising incidence rates and challenges in the effective management and treatment of diagnosed patients (Cheng et al., 2022).

The common antihypertensive drugs used in clinical practice include calcium channel blockers, angiotensin-converting enzyme inhibitors, angiotensin receptor antagonists, diuretics, β-receptor blockers, and combinations of these medications (Parodi et al., 2024). However, adverse reactions and genetic variations between individuals can affect the effectiveness of blood pressure control in hypertension patients (Johnson, 2012). Personalized diagnosis and treatment based on pharmacogenomics can effectively improve blood pressure management. Previous studies have shown that pharmacogenomics-guided medication strategies are more effective than conventional treatment approaches. Rabia et al. found that amlodipine and hydrochlorothiazide were more effective in controlling blood pressure when guided by pharmacogenomics (Johnson et al., 2019). The ADD1 GLY460Trp (r4961) suggests a decrease in baseline plasma renin activity, and hydrochlorothiazide treatment shows better blood pressure reduction in patients with Gly/Gly genotype (Cusi et al., 1997). *NEDD4L* is also considered a candidate gene influencing the response to hydrochlorothiazide. A study has shown that the antihypertensive response to hydrochlorothiazide and β-receptor blockers is better in patients with the G allele of rs4149601 compared to those with the A allele (Svensson-Färbom et al., 2011).

Despite significant progress in clinical applications of pharmacogenomics in oncology and anticoagulant therapy, its use in hypertension treatment remains limited (Cunningham and Chapman, 2019). In many cases, hypertension may not cause serious or fatal side effects, and the combination of poor patient compliance and high treatment costs has led to a lack of attention to personalized medication treatment for hypertension. Therefore, the collection and presentation of cases involving Chinese hypertensive patients benefiting from pharmacogenomics are important role for advancing personalized and precise treatment of cardiovascular diseases.

In this report, the patient presented with multiple diseases, including acute cerebral infarction, pulmonary infection, high risk of hypertension (grade 3), type 2 diabetes with poor blood glucose control, hyperhomocysteinemia, hyperlipidemia, and fatty liver. During the hypertension management period, the patient was also prescribed medications to control these complications, which were effectively managed. Controlling the patient’s blood pressure is crucial to the overall treatment process. In the early stage, the patient received a combination therapy consisting of angiotensin receptor antagonists, such as sacubitril valsartan sodium tablets (100 mg, twice daily), β-receptor blockers like metoprolol tartrate tablets (25 mg, twice daily), and calcium channel blockers, such as nifedipine controlled release tablets (30 mg, once daily). While diastolic blood pressure improved significantly, systolic blood pressure remained inadequately controlled. Potential reasons for this may include poor patient response to certain antihypertensive drugs, inappropriate dosing, or other complications that hinder effective systolic blood pressure control. However, without pharmacogenomic testing, we could only speculate on the cause and make repeatedly medication adjustments.

Pharmacogenomics testing results indicated that the genotypes of *ADRB1* and *CYP2D6* supported the recommended use for metoprolol. Sacubitril valsartan sodium tablets, a novel combination of sacubitril and valsartan in a 1:1 M ratio, were also deemed suitable for patient due to the presence of rs1283807 *ABCC9* is G/G genotype, which is associated with a factorable therapeutic response to valsartan (Kamide et al., 2013). However, the impact of CYP3A5 mutations on calcium channel blocker efficacy remains controversial. Previous report has showed that the CYP3A5 * 3 (T > C) allele is associated with an enhanced antihypertensive response to amlodipine in the Chinese population (Huang et al., 2017; Zhang et al., 2014). Nonetheless, no such correlation has been observed among Koreans and African Americans (Kim et al., 2006; Bhatnagar et al., 2010). Additionally, a study on the pharmacokinetics of nifedipine in healthy Chinese volunteers showed that CYP3A5*3 is associated with decreased nifedipine metabolism (Wang et al., 2015), although its therapeutic effects on nifedipine have not yet been fully reported.

Given the poor control of systolic blood pressure in this patient, we decided to replace nifedipine with amlodipine, as recommended by the pharmacogenomics testing. The results showed improved control of systolic blood pressure, indicating that amlodipine was more effective for this patient than nifedipine.

We initially added hydrochlorothiazide based on the patient’s *NEDD4L* and *ADD1* genotype, which may have contributed to the successful control of systolic blood pressure. Potassium ions are crucial in maintaining normal blood pressure and cardiovascular function by regulating intracellular water and ion balance, which is vital for myocardial, vascular, and renal function (Sica et al., 2002). We speculate that during the medication adjustment process, inadequate control of potassium ion concentration may have led to poor diastolic blood pressure control. Although the *ADD1* genotype suggested that spironolactone treatment should be approached with caution, we administered a low-dose of spironolactone for observation. Unfortunately, this adjustment led to a rise in systolic blood pressure and a failure to control diastolic blood pressure.

Despite the patient’s potassium ion level being below the standard range, we reverted to the original recommendations based on pharmacogenomics testing, which controlled the blood pressure at 129/90 mmHg. However, we were still unable to achieve adequate control of diastolic blood pressure. Pharmacogenomics played an important role in the series of drug adjustments, but it is disappointing that we could not achieve optimal diastolic blood pressure control. Other factors, such as medication adherence, dietary changes, and the treatment of other complications, may also affect the patient’s response to medication.

The National Guidelines for Rational Medication Use of Hypertension in China (version 2, 2017) emphasize that precision medication should consider individual genotypes to prevent inappropriate drug use and ensure personalized medication. It is generally suggested that patients undergo PGx testing before initiating relevant drugs. In clinical practice, PGx testing is also suggested when the treatment effect is unsatisfactory; however, the decision to undergo PGx testing depends on the patients’ willingness. In this case report, the patient only underwent PGx testing after poor hypertension control was observed. Although hypertension was managed through medication adjustments based on PGx testing results, the clinical use of spironolactone during the treatment process did not align with PGx recommendations, indicating that clinical decisions are not solely guided by PGx results.

Regarding the treatment of hypertension, the National Guidelines for Hypertension Management in China (2019) and the Clinical Practice Guidelines for Hypertension Management in China (2022) have not yet included specific regulations on the use of PGx testing. These clinical scenarios suggest that the guiding role of PGx testing in clinical practice requires further validation through large-scale clinical studies to accumulate more evidence in the future.

In summary, this case report demonstrated that a pharmacogenomic-guided medication strategy is effective in controlling blood pressure, preventing cardiovascular and other complications, and improving quality of life. The advancement of pharmacogenomics will help patients by reducing the discomfort associated with medication adjustments during treatment and by being more cost-effective, thereby promoting the development of personalized and precise diagnosis and treatment.

## Data Availability

The original contributions presented in the study are included in the article/Supplementary Material, further inquiries can be directed to the corresponding author.
